# Preventive Effect of Upland Pigmented Potatoes Against LPS‐Induced Inflammation in THP‐1 Macrophages

**DOI:** 10.1002/mnfr.70073

**Published:** 2025-04-25

**Authors:** Marta Toccaceli, Alessandra Marinelli, Federico Ballabio, Laura Bassolino, Roberto Lo Scalzo, Bruno Parisi, Daniela Pacifico, Federica Nicoletti, Carlo Camilloni, Giuseppe Mandolino, Katia Petroni

**Affiliations:** ^1^ Department of Biosciences Università degli Studi di Milano Milano Italy; ^2^ Research Centre for Cereal and Industrial Crops (CREA‐CI) Council for Agricultural Research and Economics Bologna Italy; ^3^ Research Centre for Engineering and Agro‐Food Processing (CREA‐IT) Council for Agricultural Research and Economics Milano Italy

**Keywords:** anthocyanins, carotenoids, chlorogenic acid, chronic inflammation, prevention

## Abstract

Aim of this study was to compare the in vitro anti‐inflammatory activity of three commercial potato varieties cultivated upland, Kennebec, Desirée, and Bleuet, whose extracts,   based on chemical analyses, were considered chlorogenic acid (CGA)‐, carotenoid‐, and anthocyanin‐rich, respectively. To this aim, THP‐1‐derived macrophages were pretreated with extracts and then challenged with LPS. While at supraphysiological doses (50 µM), all three extracts significantly counteracted LPS‐induced TNF‐α, IL‐1β, and IL‐6, at more physiologically relevant doses (1–5 µM), only Desirée and Bleuet showed anti‐inflammatory activity. We hypothesized that the high CGA content in Bleuet extract might interfere with anthocyanins. Supporting this, adding CGA to pure pelargonidin 3‐glucoside and peonidin 3‐glucoside reduced their anti‐inflammatory activity. Similarly, the isolated anthocyanin fraction from Bleuet (ACN fraction) exhibited stronger anti‐inflammatory effects than the whole Bleuet extract, and the addition of CGA to the ACN fraction significantly reduced its anti‐inflammatory effect. Molecular docking simulations suggested that glucose transporters GLUT‐1, GLUT‐3, and SGLT‐1 could be involved in this antagonist‐like interaction. In conclusion, extracts from pigmented varieties Desirée and Bleuet counteracted LPS‐induced inflammation in THP‐1 macrophages at plasma‐relevant doses. Furthermore, CGA could compete with anthocyanins for glucose transporters, limiting their cellular uptake and, consequently, their anti‐inflammatory activity.

## Graphical abstract

The anti‐inflammatory activity of extracts from three commercial potato varieties cultivated upland was investigated on THP‐1 macrophages insulted with LPS. While the chlorogenic acid (CGA)‐rich extract from Kennebec reduced inflammation only at supraphysiological doses, carotenoid‐rich Desirèe and anthocyanin (ACN)‐rich Bleuet decreased TNF‐α, IL‐1β, and IL‐6 at plasma‐relevant doses. In Bleuet extract, the high level of CGA limited the ACN anti‐inflammatory effect. Molecular docking suggested the involvement of GLUT‐1, GLUT‐3, and SGLT‐1 in this antagonist‐like effect.

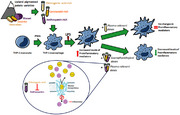

**Abbreviations**
A‐Mal: A‐maltose
ACN: anthocyanin
β‐NG: nonyl‐β‐D‐glucopyranoside
CGA: chlorogenic acid
GAPDH: glyceraldehyde‐3‐phosphate dehydrogenase
GLUT: glucose transporter
NCDs: noncommunicable diseases
Pg3G: pelargonidin 3‐glucoside
Pn3G: peonidin 3‐glucoside
SGLT: sodium/glucose cotransporter
TNF: tumor necrosis factor

## 1 Introduction

While inflammation has a beneficial and protective role in physiological conditions, by eliminating pathogens or noxious endogenous stimuli and resolving damages, chronic inflammation is a prolonged systemic inflammatory condition, recognized as a key risk factor in the onset and/or progression of various noncommunicable diseases (NCDs) [1]. These include cardiovascular, neurodegenerative, and respiratory disorders as well as diabetes and cancer, which collectively represent the leading causes of mortality and morbidity globally [2, 3]. Among the environmental and lifestyle factors promoting chronic inflammation, an unhealthy diet is one of the major contributors as well as the most preventable [4, 5]. Consequently, dietary strategies to prevent the progression of inflammation into a chronic state are attracting great interest, since the consumption of a balanced diet, rich in fruits and vegetables, has been inversely correlated with the incidence of many inflammatory‐related chronic diseases [5, 6].

Nevertheless, climate change, recently defined as “the biggest global health threat of the 21^st^ century,” is significantly affecting food production, supply, and security, as well as directly or indirectly human health, due to the effects on the availability, cost, and nutritional composition of crops [7, 8, 9]. It has been estimated that by 2050, the majority of global deaths related to climate change will be mainly attributable to changes in fruit and vegetable consumption [10]. Thus, a key challenge is to identify resilient, sustainable, and cost‐effective crops capable of growing with fewer resources and providing macro‐ and micronutrients as well as health‐promoting bioactive compounds.

Potato (*Solanum tuberosum* L.) is the third most important staple food crop after cereals [11] and represents a valuable candidate, being available in several dozen cultivated varieties in different climates and farming systems and thanks to its high nutritional value, providing energy, carbohydrates, vitamins B, C, and some minerals. The most commonly cultivated potato varieties are white‐fleshed, which are rich in phenolic acids, particularly 5‐O‐caffeoylquinic acid, commonly known as chlorogenic acid (CGA), which is reported to have several beneficial effects, including antioxidant, antidiabetic, anti‐obesity, anticancer, anti‐inflammatory, and anti‐microbial properties [12, 13]. On the other hand, pigmented varieties are also enriched in bioactive compounds, like carotenoids and anthocyanins [14, 15]. Carotenoids are vitamin A precursors and potent antioxidants, whose consumption has been inversely correlated with the incidence of many chronic diseases, including obesity, diabetes, and cancer [16], while anthocyanins have demonstrated preventive effects against cardiovascular and neurodegenerative diseases, obesity, diabetes, inflammatory and oxidative stress, and cancer [17].

Studies aimed at determining the biological activities of pigmented potato varieties are mostly limited to preclinical studies and mainly focused on purple/red varieties [18]. Namely, purple‐fleshed potatoes prevented gastro‐intestinal inflammation, microbiota dysfunction, and gut barrier impairment in high‐fat diet‐fed pigs [19] and dextran sodium sulfate (DSS)‐induced colitis in mice [20, 21], while reducing obesity, hyperglycemia, and oxidative stress in diabetic or hypercholesterolemic rats [22, 23, 24, 25, 26, 27]. Additionally, purple and red potatoes reduced chemically‐induced stomach and breast cancer in rats [28, 29].

A few clinical studies have demonstrated that the consumption of pigmented potatoes, both yellow‐ and purple‐fleshed, reduces inflammation and DNA damage compared to the white ones [30], whereas purple potatoes were associated with a significant decrease in blood pressure [31], postprandial glycemia and insulinemia [32, 33, 34], and arterial stiffness [35].

However, no studies have been undertaken to assess the synergistic or antagonistic effect of different classes of bioactive compounds or the combination of different polyphenols in potatoes. Furthermore, most in vitro studies on the anti‐inflammatory, antioxidative, or anticancer activity of pigmented potato extracts used supraphysiological concentrations (e.g., 10–100 µM or higher for anthocyanins) [36, 37]. In contrast, plasma‐relevant concentrations (e.g., 0.1–10 µM for anthocyanins) should be used in order to determine mechanisms of action and health‐promoting effects under more physiologically‐related experimental conditions [38, 39].

This work aimed at evaluating and comparing the anti‐inflammatory activity of extracts obtained from tubers of three commercial potato varieties named Kennebec (yellow skin and white flesh), Desirée (red skin and yellow flesh), and Bleuet (purple skin and flesh) from upland cultivation in THP‐1‐derived human macrophages insulted with LPS. The characterization of extracts allowed us to consider Kennebec, Desirée, and Bleuet varieties as differently enriched in CGA, carotenoids, and anthocyanins. The dose‐dependent activity of plasma‐relevant concentrations of the three extracts, the anthocyanin fraction of Bleuet, or the combination of pure compounds against LPS‐induced pro‐inflammatory cytokine (TNF‐α, IL‐1β, IL‐6) genes and proteins has been evaluated.

## 2 Experimental Section

### 2.1 Materials

CGA, pelargonidin 3‐glucoside (Pg3G), and peonidin 3‐glucoside (Pn3G) used for cell treatments were purchased at Extrasynthese (Genay, France). Human leukemia‐derived THP‐1 monocytes (ATCC‐TIB‐202) were purchased from LGC Standards S.r.L. (Milan, Italy).

### 2.2 Potato Tubers

**FIGURE 1:**
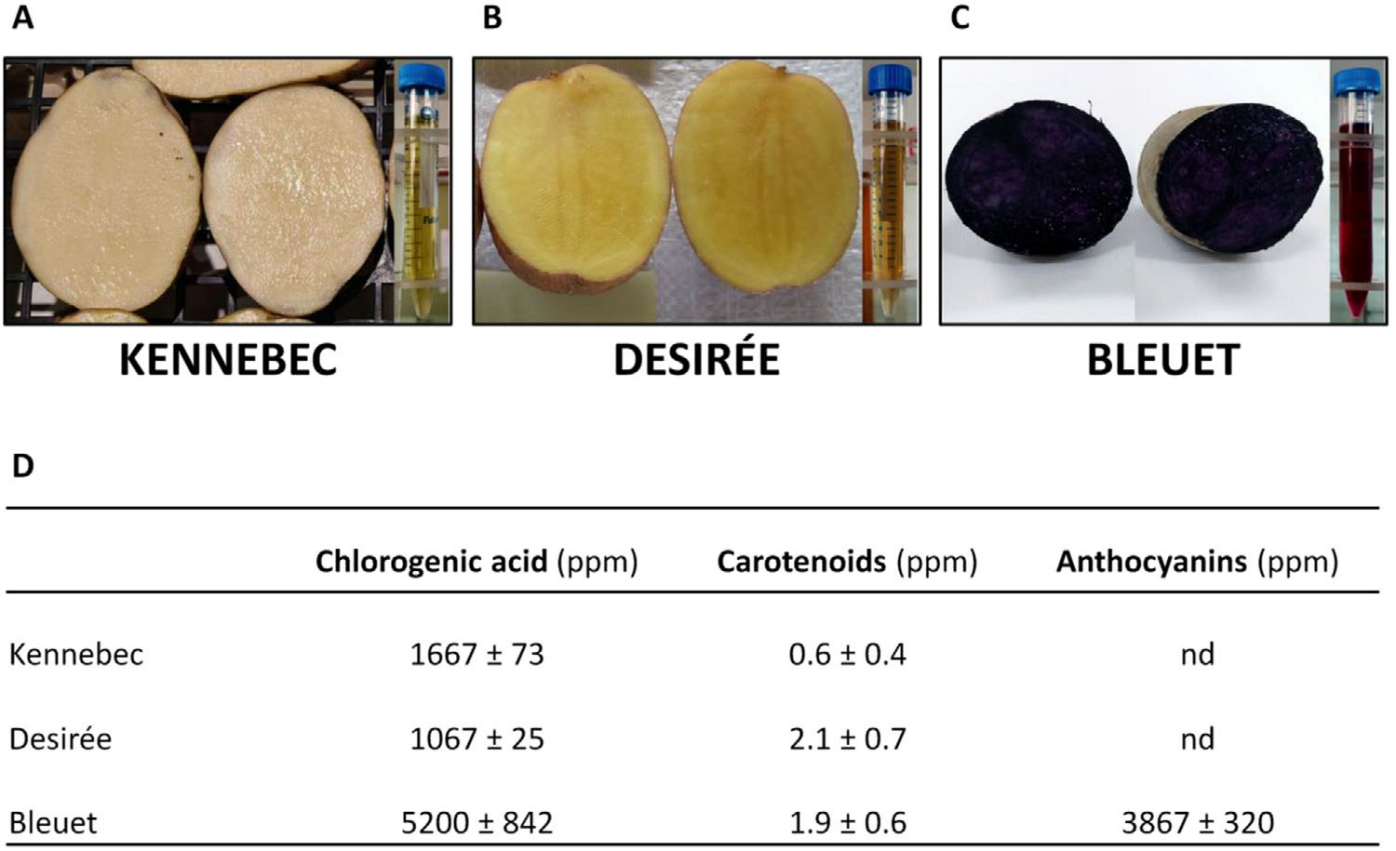
Upland potato tubers and extracts (A–C) and the phytonutrient content in potato extracts (D). Values are the average (±SD) of three replicates and are expressed as ppm (mg/L) of CGA, of zeaxanthin equivalents for carotenoids, and Pg3G equivalents for anthocyanins, respectively. CGA = chlorogenic acid, nd = not detected.

Three commercial potato varieties were selected: the yellow‐skinned and white‐fleshed cv. Kennebec (Figure 1A), the red‐skinned and yellow‐fleshed cv. Desirée (Figure 1B), and the dark purple‐skinned and ‐fleshed Bleuet (Figure 1C). All the potato varieties were cultivated under local agronomic practices in the experimental field located in Starleggia (Campodolcino, Sondrio Province, Spluga Valley, Lombardy Region, Italy) at 1560 masl. The tubers were harvested during the first week of October 2019 at the senescence growth stage BBCH 97 907 [40], that is, leaves and stem dead, stems bleached and dry. For each genotype, at least a bulk of 100 US No. 1 tubers (5 cm diameter or weighing a minimum of 112 g) was selected from randomized plots (5 tubers per 5 single plants per 4 replications per single variety) and lyophilized for subsequent extraction procedures.

### 2.3 Extraction and Fractionation

Extracts used for the in vitro cell experiments were obtained from lyophilized whole potato tubers (peel and flesh) of Kennebec, Desirée, and Bleuet varieties (Figures 1A–C), as described in Supplementary Methods. The anthocyanin fraction (ACN fraction) was isolated from Bleuet extract via liquid‐liquid extraction using a separatory funnel, as described in Supplementary Methods.

### 2.4 Spectrophotometric and HPLC‐DAD Analyses of Extracts and ACN Fraction

Potato extracts and the ACN fraction were analyzed and characterized using spectrophotometry and HPLC‐DAD to determine their content of CGA, carotenoids, and anthocyanins, as described in Supplementary Methods.

### 2.5 Cell Culture, Cytotoxicity Assay, and Treatments

Human leukemia‐derived THP‐1 monocytes were cultured in RPMI 1640 culture medium (EuroClone, Pero, MI, Italy) supplemented with 10% fetal bovine serum (FBS, Sigma‐Aldrich, St. Louis, MO, USA), 2 mM L‐glutamine (Sigma‐Aldrich), 100 units/mL penicillin, and 100 µg/mL streptomycin (Gibco/BRL, Carlsbad, CA, USA) at 37°C in a humidified atmosphere containing 5% CO_2_. In order to differentiate THP‐1 monocytes into adherent macrophages, 2 × 10^6^ cells/well were seeded in 12‐well plates with 25 ng/mL Phorbol 12‐Myristate 13‐Acetate (PMA, Sigma‐Aldrich) for 24 h. Non‐cytotoxicity was verified by treating THP‐1 macrophages for 48 h with different concentrations of Kennebec, Desirée, and Bleuet extracts (0, 5, 25, 50, 75 µM). Concentrations were determined based on the Pg3G equivalent content of the Bleuet extract or ACN fraction, and the other extracts were diluted in culture medium using the same volumes. Cell viability was determined using the 3‐(4,5‐dimethylthiazol‐2‐yl)‐2,5‐diphenyltetrazolium bromide (MTT, Sigma‐Aldrich) test as previously described [41] and the lactate dehydrogenase (LDH) activity assay (Sigma‐Aldrich) following the manufacturer's instructions.

To mimic a preventive treatment, THP‐1 macrophages were then pretreated with or without potato extracts (1–50 µM), ACN fraction (1–5 µM), or pure compounds (1–10 µM) for 16 h before inflammatory stimulus. To induce a pro‐inflammatory response, cells were co‐treated with or without 1 µg/mL LPS (Sigma‐Aldrich) for 6 h for real time RT‐PCR analysis or 24 h for ELISA assays, in the presence or absence of potato extracts, ACN fraction, or pure compounds. Time points were chosen based on previous setup experiments, since 6 h of LPS treatment showed the highest transcript levels of pro‐inflammatory cytokine genes, whereas we observed the highest levels of cytokine secretion after 24 h of LPS treatment (data not shown).

### 2.6 RNA Extraction and Real Time RT‐PCR Analysis

Total RNA was isolated from cells, reverse‐transcribed to cDNA, and used to determine transcript levels using gene‐specific primers (Table S1) as described in Supplementary Methods.

### 2.7 Enzyme‐Linked Immunosorbent Assay (ELISA)

Cytokine concentrations in cell supernatants were detected using Human TNF‐α, Human IL‐1β and Human IL‐6 DuoSet ELISA Kits (R&D Systems, Minneapolis, MN, USA), as described in Supplementary Methods.

### 2.8 Molecular Docking

The docking pipeline was performed using the Schrödinger Maestro release 2023‐3 build 125 (Maestro, Schrödinger, LLC, New York, NY, 2023). The crystallographic structures of human glucose transporter type 1 (GLUT‐1), type 3 (GLUT‐3), and sodium/glucose cotransporter 1 (SGLT‐1) were retrieved from the Protein Data Bank [42]. Specifically, the inward‐open conformation of GLUT‐1 bound to nonyl‐β‐D‐glucopyranoside (β‐NG; PDB ID: 4PYP), the outward‐open conformation of GLUT‐3 bound to A‐maltose (A‐Mal; PDB ID: 4ZWC), and the partial inward‐open apo conformation of SGLT‐1 (PDB ID: 7SLA) were selected (Figure S4). Ligands and water molecules located more than 5 Å from the ligands were removed. Structural refinement was performed as described in the Supplementary Methods. The putative ligands Pg3G, Pn3G, and CGA were reconstructed from the SMILES strings associated with PubChem CIDs (443648, 443654, and 1794427, respectively). Ligand geometries were optimized using the LigPrep tool [43], and protonation states at pH 7 were generated using Epik [44].

Docking grids were designed for each transporter to include the entire substrate translocation channel, as described in Supplementary Methods.

The best three different docking poses for each ligand were analyzed for their interactions with the target proteins. The docking process and the bidimensional protein‐ligand interaction diagrams were performed as described in the Supplementary methods.

The target proteins and the best docking poses are available on Zenodo [45] (https://doi.org/10.5281/zenodo.14681251).

### 2.9 Statistical Analysis

Data were analyzed through one‐way ANOVA followed by Tukey's post hoc test for multiple comparisons using GraphPad Prism 8 software. Differences with *p* < 0.05 were considered significant.

## 3 Results

### 3.1 Phytonutrient Composition of the Upland Potato Extracts

The composition of the extracts from the three selected potato varieties, Kennebec, Desirée, and Bleuet (Figures 1A–C), in total CGA, carotenoids, and anthocyanins was analyzed by spectrophotometry and HPLC‐DAD. Figure 1D shows that anthocyanins were only present in Bleuet extract (3867 ± 320 ppm), carotenoids were present at comparable levels in Bleuet and Desirée (1.9 ± 0.6 and 2.1 ± 0.7 ppm, respectively), and at 3‐fold lower levels in Kennebec (0.6 ± 0.4 ppm), whereas CGA was present at comparable levels in Desirée and Kennebec (1067 ± 25 and 1667 ± 73 ppm, respectively), but at significantly higher levels in Bleuet (5200 ± 842 ppm). Based on these data, Kennebec, Desirée, and Bleuet extracts were considered CGA‐rich, carotenoid‐rich, and anthocyanin‐rich based on their distinctive phytonutrients, respectively (Figure 1D).

### 3.2 Desirée and Bleuet Extracts Are More Effective at Plasma‐Relevant Doses Compared to Kennebec

**FIGURE 2:**
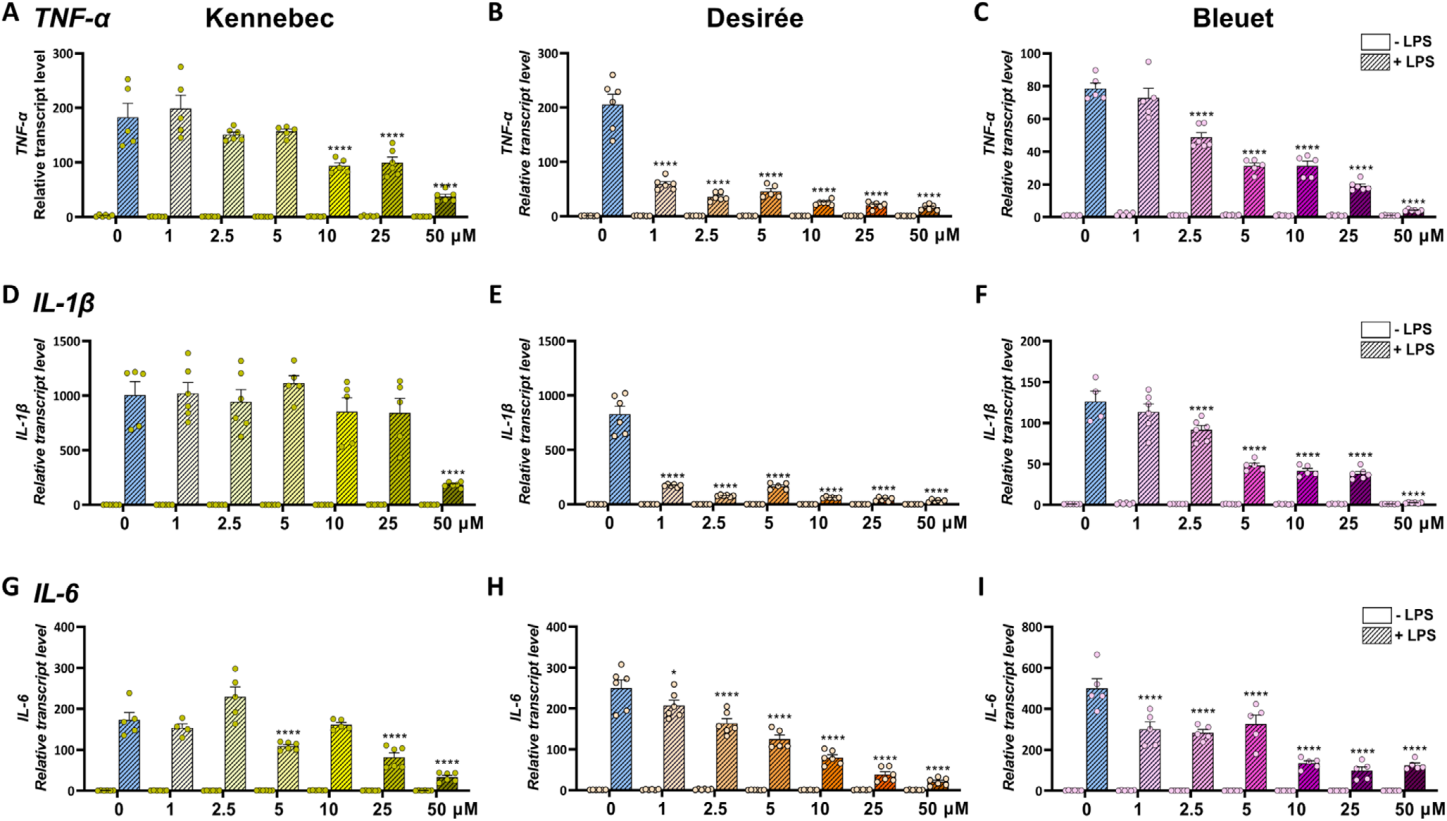
Dose‐response analysis of the anti‐inflammatory activity of Kennebec, Desirée, or Bleuet extract. qRT‐PCR analysis of pro‐inflammatory *TNF‐α* (A–C), *IL‐1β* (D–F), and *IL‐6* (G–I) genes in THP‐1 macrophages pretreated for 16 h with increasing concentrations (0, 1, 2.5, 5, 10, 25, 50 µM) of potato extracts from Kennebec (A, D, G), Desirée (B, E, H), or Bleuet (C, F, I) without LPS (−LPS) and then co‐treated for 6 h with extracts and 1 µg/mL LPS (+LPS). Each transcript level was normalized against GAPDH and expressed as a fold change over the unchallenged counterpart (−LPS). Data are presented as mean ± SEM of two biological replicates in triplicate (*n* = 4–6). *p < 0.05, ****p < 0.0001 indicate significant differences versus 0 µM + LPS.

**FIGURE 3:**
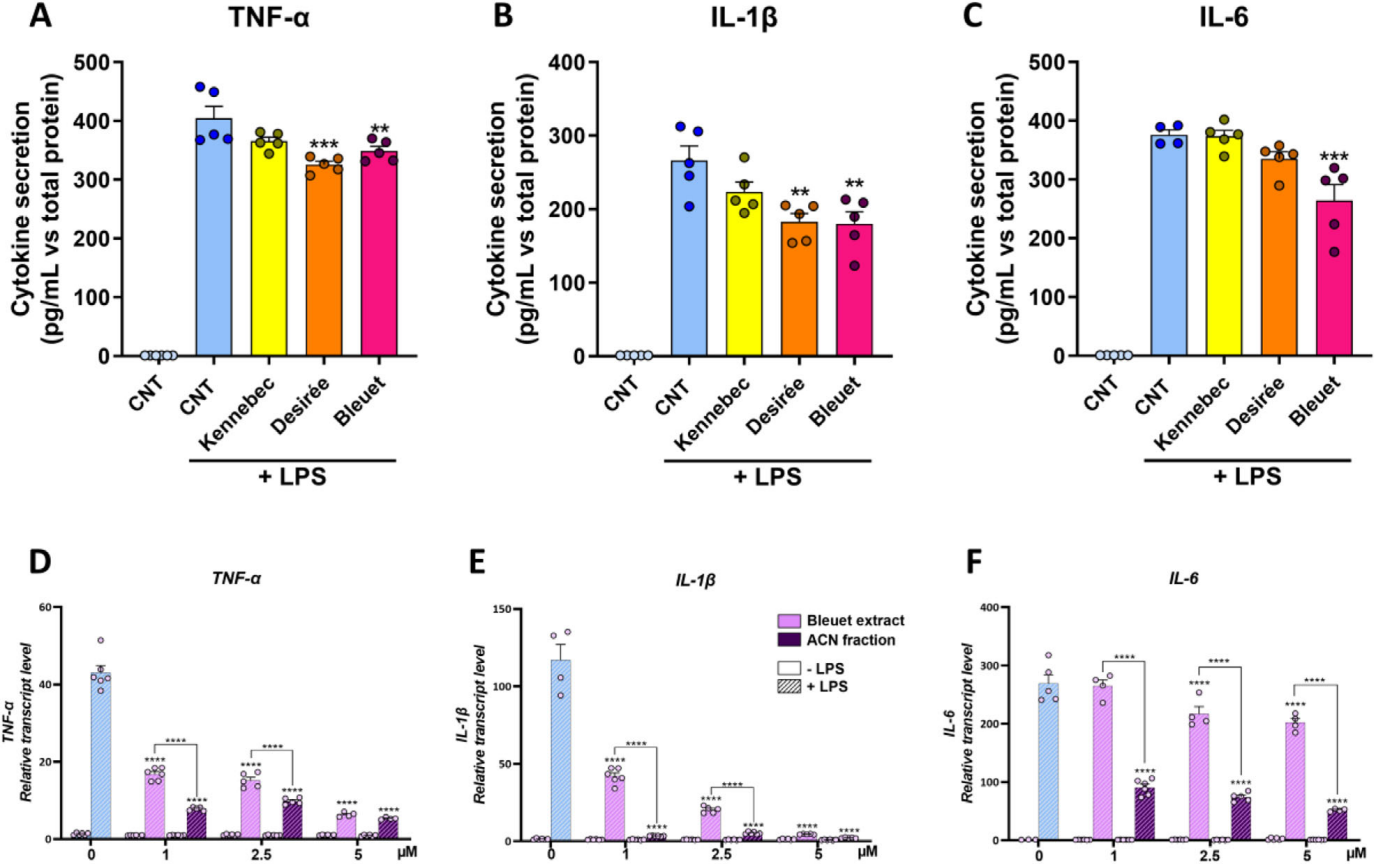
Effect of a plasma‐relevant dose treatment of potato extracts against LPS‐induced cytokine secretion (A–C) and qRT‐PCR analysis of transcript levels of pro‐inflammatory genes after treatment with Bleuet extract versus its isolated ACN fraction (D–F). ELISA assay of pro‐inflammatory TNF‐α (A), IL‐1β (B), and IL‐6 (C) cytokines secreted in the medium by THP‐1 macrophages pretreated for 16 h without (CNT) or with 5 µM of Kennebec, Desirée, or Bleuet extracts without LPS and then co‐treated for 24 h with extracts and 1 µg/mL LPS (+LPS). Data were normalized against the total protein (µg) value of each sample and expressed as mean ± SEM (pg/mL vs. µg total protein). Analysis was conducted in duplicate with four technical replicates (*n* = 4–6) (A–C). qRT‐PCR analysis of *TNF‐α* (D), *IL‐1β* (E), and *IL‐6* (F) transcript levels in THP‐1 macrophages pretreated for 16 h with increasing concentrations (0, 1, 2.5, 5 µM) of Bleuet extract or ACN fraction without LPS (−LPS) and then co‐treated for 6 h with extracts/ACN fraction and 1 µg/mL LPS (+LPS). Each transcript level was normalized against GAPDH and expressed as a fold change over the unchallenged counterpart (−LPS). Data are presented as mean ± SEM of two biological replicates in triplicate (*n* = 4–6) (D–F). **p < 0.01, ***p < 0.001, ****p < 0.0001 indicate significant differences versus CNT + LPS (A–C) or 0 µM + LPS and noted pairs (D–F). ELISA, enzyme‐linked immunosorbent assay.

To compare the anti‐inflammatory efficacy of the different potato extracts, the transcript levels of pro‐inflammatory genes (*TNF‐α*, *IL‐1β*, *IL‐6*) were analyzed by qRT‐PCR (Figure 2). THP‐1 macrophages were treated with increasing concentrations of extracts up to 50 µM, which was proven to be non‐cytotoxic up to 48 h (Figure S3). Treatment with CGA‐rich extract from Kennebec reduced the LPS‐induced transcript levels of pro‐inflammatory cytokine genes only at supraphysiological concentrations (10‐50 µM for *TNF‐α* and *IL‐6*; only 50 µM for *IL‐1β*) (Figures 2A,D,G). On the contrary, treatment with carotenoid‐rich Desirée and anthocyanin‐rich Bleuet extracts significantly reduced their expression in a dose‐dependent manner starting from 1–2.5 µM, with a stronger reduction of *TNF‐α* and *IL‐1β* observed for Desirée (Figures 2B,E) compared to Bleuet (Figures 2C,F), whereas they determined a comparable reduction in the transcript level of *IL‐6* (Figures 2H–I) with respect to LPS treatment alone (0 μΜ + LPS). These data were supported by ELISA analysis of pro‐inflammatory cytokines secreted in the cell medium after the treatment with 5 μΜ potato extracts (Figures 3A–C). Kennebec extract did not show a significant anti‐inflammatory effect, as it did not reduce TNF‐α, IL‐1β, nor IL‐6 secretion compared with LPS treatment alone (CNT + LPS), while Desirée and Bleuet extracts counteracted LPS‐induced TNF‐α and IL‐1β (Figures 3A–B) secretion in a comparable way (−15%–18% and −31%–33%, respectively), but only the anthocyanin‐rich extract from Bleuet significantly reduced LPS‐induced IL‐6 secretion (−27%, Figure 3C).

### 3.3 Effect of Bleuet Extract Versus the Isolated ACN Fraction on Transcript Levels of Pro‐Inflammatory Cytokines

Considering that anthocyanins are well known for their direct anti‐inflammatory activity, whereas carotenoids are more commonly associated with antioxidant properties [16], we hypothesized that some other compounds in Bleuet extract may interfere with anthocyanins in counteracting LPS‐induced inflammation. To explore this hypothesis, the effect of the isolated anthocyanin fraction from Bleuet extract (ACN fraction) versus Bleuet extract on transcript levels of pro‐inflammatory cytokines was analyzed up to 5 µM treatment (Figures 3D–F). Though the treatment with Bleuet extract was able to reduce *TNF‐α*, *IL‐1β*, and *IL‐6* transcripts, the treatment with the isolated ACN fraction was always more effective in lowering them. This difference was particularly evident for *IL‐6*, which was strongly reduced by the ACN fraction with respect to the Bleuet extract at all the concentrations tested (*p <* 0.0001, Figure 3F). For *TNF‐α* and *IL‐1β* (Figures 3D–E), the anti‐inflammatory effect of the isolated ACN fraction was significantly more potent than the Bleuet extract up to 2.5 µM treatment, whereas at 5 µM the reduction was stronger, but not significantly different compared to Bleuet extract.

### 3.4 Anti‐Inflammatory Activity of the Isolated ACN Fraction From Bleuet May Be Inhibited by CGA

**FIGURE 4:**
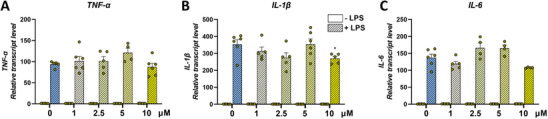
Dose‐response analysis of the anti‐inflammatory activity of CGA. qRT‐PCR analysis of transcript levels of pro‐inflammatory *TNF‐α* (A)*, IL‐1β* (B), and *IL‐6* (C) genes in THP‐1 macrophages pretreated for 16 h with increasing concentrations of CGA (0, 1, 2.5, 5, 10 µM) without LPS (−LPS) and then co‐treated for 6 h with CGA and 1 µg/mL LPS (+LPS). Each transcript level was normalized against GAPDH and expressed as a fold change over the unchallenged counterpart (−LPS). Data are presented as mean ± SEM of two biological replicates in triplicate (*n* = 4–6). *p < 0.05 indicates a significant difference versus 0 µM + LPS.

Considering that the CGA‐rich extract from Kennebec (Figures 2A,D,G) and similarly pure CGA (Figure 4) did not show an anti‐inflammatory effect at plasma‐relevant doses, and that the Bleuet extract was particularly enriched in CGA (Figure 1D), we investigated the possible involvement of this compound in interfering with the anti‐inflammatory activity of anthocyanins.

**FIGURE 5:**
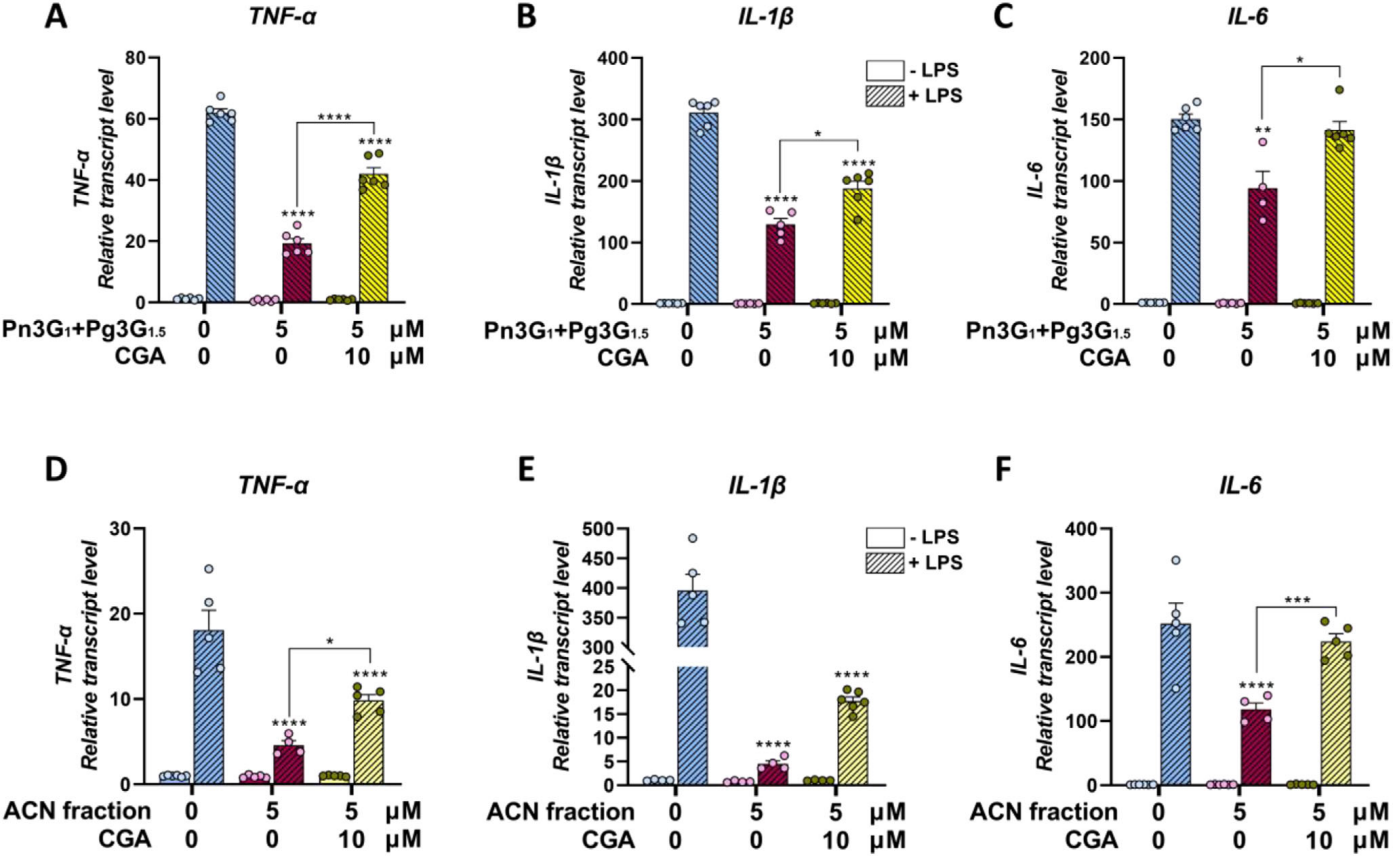
qRT‐PCR analysis of pro‐inflammatory *TNF‐α* (A, D), *IL‐1β* (B, E), and *IL‐6* (C, F) genes upon treatment with pure anthocyanins (A–C) or ACN fraction (D–F) with or without CGA. THP‐1 macrophages were pretreated for 16 h in the absence (0 µM) or presence of a 5 µM mixture of pure Pn3G and Pg3G (1:1.5 ratio) (A–C) or a 5 µM ACN fraction (D–F) alone or combined with 10 µM pure CGA without LPS (−LPS) and then co‐treated for 6 h with pure compounds or ACN fraction and 1 µg/mL LPS (+LPS). Each transcript level was normalized against GAPDH and expressed as a fold change over the unchallenged counterpart (−LPS). Data are presented as mean ± SEM of two biological replicates in triplicate (*n* = 4–6). *p < 0.05, **p < 0.01, ***p < 0.001, ****p < 0.0001 indicate significant differences versus 0 µM + LPS and noted pairs.

Firstly, the effect of a 5 µM 1:1.5 mixture of pure Pn3G and Pg3G, representing the ratio of the main anthocyanins in Bleuet extract, on transcript levels of pro‐inflammatory cytokine genes was verified (Figures 5A–C). All the LPS‐induced genes tested were significantly reduced by this mixture alone, but when 10 µM CGA was added, this anti‐inflammatory effect was reduced for *TNF‐α* and *IL‐1β*  (Figures 5A–B) or completely lost for *IL‐6* (Figures 5C). The concentration of CGA has been chosen to maintain a ratio between anthocyanins:CGA similar to the one in Bleuet extract.

Similarly, to better mimic the composition of Bleuet extract, THP‐1 macrophages were treated with 5 µM ACN fraction alone or in combination with 10 µM pure CGA (Figures 5D–F). Results showed that, also in this case, while the ACN fraction significantly downregulated LPS‐induced pro‐inflammatory gene expression (*p <* 0.0001), CGA reduced its anti‐inflammatory activity. In particular, the addition of CGA to the ACN fraction resulted in a less severe down‐regulation of *TNF‐α* and *IL‐1β* genes compared to the ACN fraction alone, but still significant compared to LPS treatment alone (*p* < 0.0001, Figures 5D–E), and a complete loss of effectiveness in lowering *IL‐6* (Figure 5F). Despite the difference in *IL‐1β* transcript levels between the ACN fraction alone and in combination with CGA was not statistically significant, an increasing trend was observed (Figure 5E).

### 3.5 Putative Involvement of Glucose Transporters by Molecular Docking

Anthocyanins are reported to be taken up by different cell types also through glucose transporters, thanks to their sugar moiety, which binds to these proteins and facilitates their transport [46, 47]. On the other hand, CGA is known to regulate glucose metabolism [12], and interestingly, the quinic acid molecule in its chemical structure resembles glucose. Therefore, we hypothesized that CGA might compete with anthocyanins for glucose transporters, limiting their uptake. To explore this hypothesis, we used molecular docking.

**FIGURE 6:**
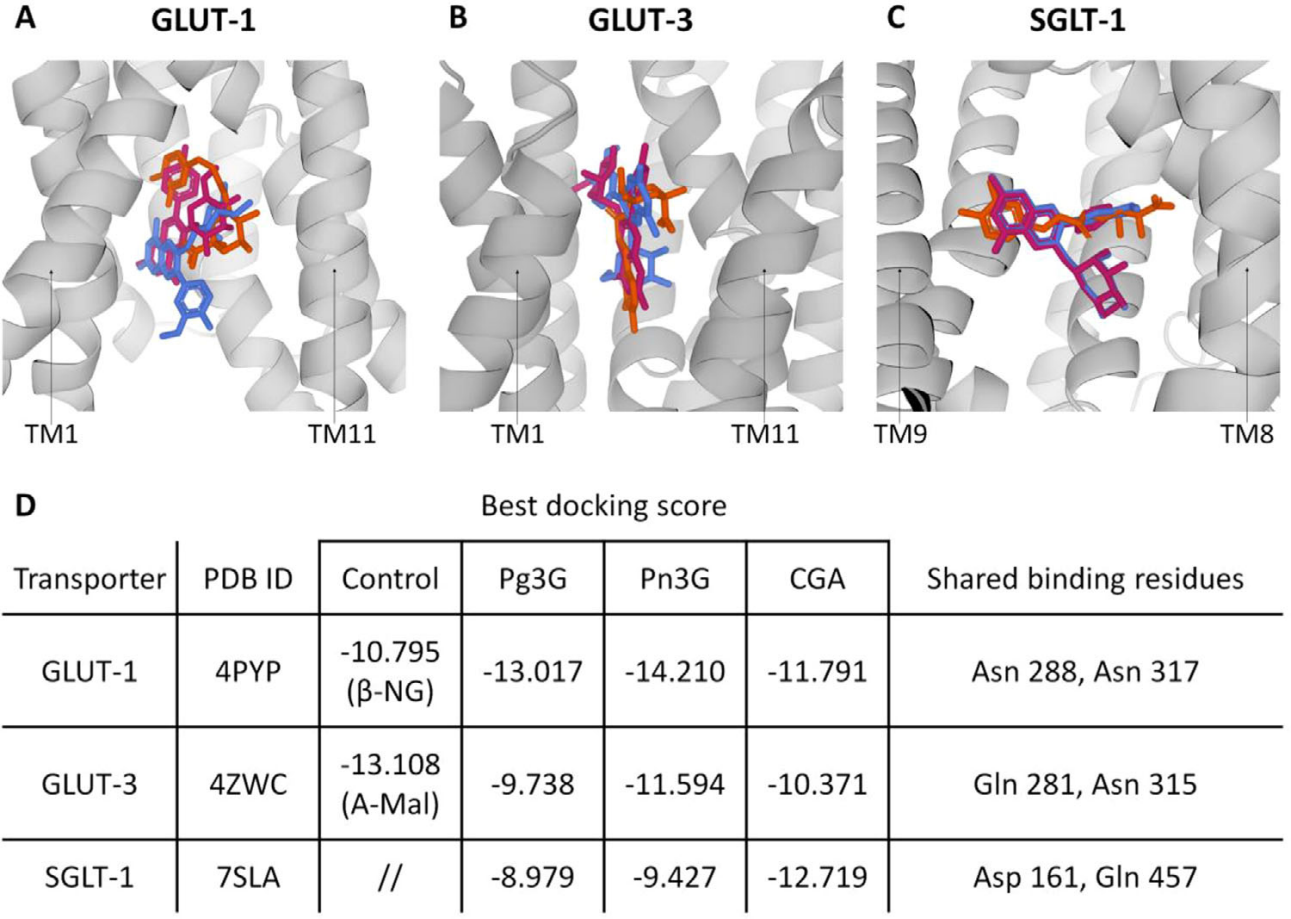
Molecular Docking. Best docking poses of Pg3G, Pn3G, and CGA in GLUT‐1 (A), GLUT‐3 (B), and SGLT‐1 (C). The ligands are superimposed within each transporter channel and represented as sticks. All the atoms of Pg3G are colored in pink, Pn3G in blue, and CGA in orange. Hydrogen atoms have been omitted for clarity. GLUT‐1 and GLUT‐3 are represented in a gray cartoon. The transmembrane (TM) helices 2, 3, 4, and 6 are transparent to allow the observation of the ligands. TM1 and TM11 are indicated as references (A–B). SGLT‐1 is represented in a gray cartoon. TM2 and TM6 are transparent to allow the observation of the ligands. TM8 and TM9 are indicated as a reference (C). For each transporter, the best docking score of each ligand is reported. A residue is considered common if the interaction is shared at least by one pose for the ligand, including the control (ligand present in the x‐ray structure) (D).

The docking simulations for GLUT‐1 revealed overlaps in residues interacting with Pg3G, Pn3G, and CGA (Figure 6A). Key amino acids shared among the ligands include Asn 288, Asn 317, Trp 388, Asn 411, and Asn 415, which were observed to interact with all three ligands in at least one pose (Figure S11A). CGA showed docking scores of −11.791 (pose 1), −11.726 (pose 2), and −10.952 (pose 3), which, although less negative, are still comparable to those of the Pg3G (−13.017, −12.810, −12.750) and Pn3G (−14.210, −13.768, −13.391) (Figures S11A,S5,S6). Notably, CGA, as well as the tested anthocyanins, interacts with residues Asn 288 and Asn 317, which are also engaged by the ligand β‐NG in the x‐ray solved structure (Figures 6D, S5,S11A). β‐NG was reintroduced in GLUT‐1 with a docking score of −10.795 (Figure 6D).

For GLUT‐3, docking simulations identified significant residue overlaps among Pg3G, Pn3G, and CGA (Figure 6B). Shared residues, observed in all three ligands in at least one pose, include Asn 32, Gln 281, Asn 315, and Asn 413. The docking scores of CGA (−10.371, −9.794, −9.476) were close to those of Pg3G (−9.738, −9.305, −9.089) and Pn3G (−11.594, −11.082, −9.736) (Figures S11B,S7,S8). The three ligands engage residues that overlap with the A‐Mal molecule in the crystal structure, such as Gln 281 and Asn 315 (Figure 6D). Furthermore, all CGA poses show interactions with Gln 159, which is in common with A‐Mal but not with the other tested anthocyanins. A‐Mal was reintroduced in GLUT‐3 with a docking score of −13.108 (Figures S11B,S7).

In SGLT‐1, significant residue overlaps among Pg3G, Pn3G, and CGA were also identified by docking simulations (Figure 6C). CGA exhibited the strongest binding affinity with docking scores (−12.719, −10.476, −10.329) compared to Pg3G (−8.979, −8.816, −8.571) and Pn3G (−9.427, −9.240, −7.423) (Figures S11C,S9,S10). Interactions with Lys 157 are established in all the poses of CGA but not with the other ligands (Figure S11C). Asp 161 and Gln 457 are common across all the ligands (Figure 6D).

In general, most of the interactions established by the ligands with the transporter residues were hydrogen bonds, predominantly involving asparagine and glutamine residues, which are abundant in the substrate translocation channel of GLUT‐1, GLUT‐3, and SGLT‐1. In addition, a few π‐π stacking interactions were observed, involving tryptophan and phenylalanine residues of the transporters (Figures S6,S8,S10).

## 4 Discussion

Our study aimed at comparing the anti‐inflammatory potential of three commercial potato varieties from upland cultivation differently enriched in health‐beneficial compounds, such as CGA [12], carotenoids [16], and anthocyanins [17], in THP‐1‐derived human macrophages insulted with LPS. Based on the chemical analysis, extracts from whole tubers were considered as mainly CGA‐rich (cv. Kennebec), carotenoid‐rich (cv. Desirée), and anthocyanin‐rich (cv. Bleuet).

In this study, all potato extracts significantly reduced the transcript levels of LPS‐induced pro‐inflammatory cytokine genes at supraphysiological doses above 25–50 µM, but at doses closer to those reachable in vivo after pigmented potato consumption [48], only extracts from Desirée and Bleuet (1–5 µM) exerted an anti‐inflammatory effect. These results were confirmed by cytokine secretion analysis after the treatment with 5 μΜ potato extracts. Although our results on CGA‐rich extract from Kennebec appear to contradict studies reporting CGA as an anti‐inflammatory agent [49, 50, 51], it is important to note that most of them used supraphysiological doses, exceeding 5 µM. Our results also showed that 50 µM CGA significantly reduced inflammation, in accordance with a study reporting that 50 µM CGA counteracted LPS‐induced inflammation in RAW264.7 murine macrophages, reducing TNF‐α, IL‐6, and nitric oxide [49]. Similarly, CGA exerted anti‐inflammatory and anti‐apoptotic properties by inhibiting NF‐κB on both M13‐03 human oligodendrocyte‐like cells and HaCaT human keratinocytes when provided at doses around 100 µM [50, 51]. Nevertheless, these CGA concentrations are hardly achievable in human plasma, given the low bioavailability of CGA, around 33% [12, 52]. Investigating nutrient bioactivity at plasma‐relevant concentrations is crucial for understanding their mechanisms of action. A study on HUVEC cells reported that plasma‐relevant doses of cyanidin 3‐glucoside reduced IL‐6 levels induced by CD40L but not by oxidized LDL, which are, however, regulated by its metabolites, indicating that at these doses the anti‐inflammatory activity is regulated by different pathways [39]. In accordance with our work, 5 µM CGA alone showed no effect against LPS‐induced inflammation in THP‐1 macrophages [53]. Moreover, CGA has been recently described as a potent anti‐cancer compound able to promote macrophage polarization toward an M1 pro‐inflammatory phenotype in a tumor environment context [54, 55].

The stronger anti‐inflammatory activity of carotenoid‐rich extract from Desirée compared to Bleuet was unexpected, particularly since carotenoids are primarily recognized for their antioxidant properties [16]. Moreover, the main carotenoids in Desirée extract, violaxanthin and antheraxanthin [56], are less studied for their anti‐inflammatory properties compared to their de‐epoxy derivatives, lutein and zeaxanthin. To our knowledge, only one study has investigated the anti‐inflammatory potential of violaxanthin, reporting that significant inhibition of the LPS‐induced NF‐κB pathway activation in RAW264.7 murine macrophages required concentrations above 30–60 µM [57]. On the other hand, other xanthophylls in the Desirèe variety, such as lutein and zeaxanthin [56], have demonstrated anti‐inflammatory activity in vivo in patients with stable angina and ex vivo in peripheral blood mononuclear cells (PBMCs) from coronary artery disease patients [58]. Thus, the strong effect of the carotenoid‐rich extract from Desirèe in our study could potentially be attributed to these carotenoids. However, their concentration is very low, especially compared to the anthocyanin content in Bleuet extract used as a reference (5 µM anthocyanins corresponded to around 1.9 nM carotenoids).

The milder anti‐inflammatory effect of the anthocyanin‐rich extract from Bleuet could be due to the predominance of pelargonidin‐ and peonidin‐based anthocyanins. In fact, a study on RAW264.7 murine macrophages demonstrated that the anthocyanidin structure influences its anti‐inflammatory activity, with only delphinidin and cyanidin inhibiting the LPS‐induced cyclooxygenase‐2 (COX‐2) production, while pelargonidin, peonidin, and malvidin show no effect [59]. Moreover, we speculated that compounds other than anthocyanins in Bleuet extract might interfere with their well‐reported anti‐inflammatory activity. Comparing Bleuet extract to its isolated ACN fraction, we found that the ACN fraction showed stronger anti‐inflammatory activity at plasma‐relevant doses (1–5 µM). Since Bleuet extract was particularly enriched in CGA, which showed no anti‐inflammatory activity at plasma‐relevant doses, the possible involvement of CGA in disturbing the anthocyanin anti‐inflammatory activity was investigated firstly in combination with pure anthocyanins (Pg3G and Pn3G) and then with the isolated ACN fraction from Bleuet. Consistent with our hypothesis, in both cases, 10 µM CGA inhibited the anti‐inflammatory effect of 5 µM anthocyanins. In particular, while their activity on *TNF‐α* and *IL‐1β* was lowered in combination with CGA, the effect of anthocyanins on LPS‐induced *IL‐6* was completely lost, maintaining the *IL‐6* transcripts at the same level as the LPS treatment alone.

Regarding the mechanism by which CGA interferes with the anti‐inflammatory activity of anthocyanins, further investigations are required to deepen insights into phytonutrient interactions. However, we hypothesized the involvement of glucose transporters, since anthocyanins can also be taken up via these transporters, thanks to their sugar moiety [46, 47]. Moreover, CGA can modulate glucose metabolism, contributing to its beneficial effects against diabetes and hyperglycemia [60, 61, 62]. CGA can modulate the expression of glucose transporters, such as SGLT‐1 and GLUT‐2, thus regulating intestinal glucose absorption and homeostasis in rats under a high‐fat diet [61]. However, despite the structural similarity between the quinic acid moiety and glucose, no studies have reported CGA's direct interaction with glucose transporters. Thus, we investigated via molecular docking the possible interaction of CGA with GLUT‐1, GLUT‐3, and SGLT‐1 known to be involved in the uptake of anthocyanins [46, 47] and expressed by macrophages [63]. To comprehensively explore the binding behavior of Pg3G, Pn3G, and CGA, different transporter conditions were considered in the docking pipeline. This approach allowed the assessment of ligand binding across different states of the substrate translocation channel, providing insights into the capability of the ligands to interact with and potentially pass through the channel under different protein conditions. Our docking analysis suggests that CGA may compete with anthocyanins, specifically Pg3G and Pn3G, for binding to the glucose transporters GLUT‐1, GLUT‐3, and SGLT‐1. This potential competition arises from the ability of CGA to establish interactions with residues that are also targeted by the anthocyanins. The structural similarity in binding modes between Pg3G, Pn3G, and CGA extends beyond shared residues to their ability to occupy overlapping regions within the substrate translocation channel, mimicking each other. This spatial similarity may allow CGA to interact with key residues in a manner comparable to the anthocyanins. This behavior was observed across all three transporters, as illustrated by the docking poses and interaction networks. Hence, since glucose transporters are saturable transporters, the high levels of CGA in Bleuet extract may compete with anthocyanins for these transporters, limiting their cellular uptake and, consequently, their anti‐inflammatory activity. While other tea polyphenols have been reported to bind SGLT‐1 as antagonist‐like molecules without being transported via this transporter [64], similar studies have not yet been conducted on CGA.

Despite some limitations regarding cooking and digestion effects, our results are in accordance with a human study where, unlike white‐fleshed potatoes, consuming yellow‐ or purple‐fleshed potatoes, rich in carotenoids and anthocyanins, reduced serum IL‐6 and C‐reactive protein (CRP) levels [30].

In summary, our study suggests that pigmented potatoes from upland cultivation, such as Desirèe and Bleuet, may represent an economic resource for local growers, considering their improved health‐promoting bioactive compounds and their ability to counteract inflammatory‐related diseases. Moreover, our data provide, for the first time, the hypothesis that CGA may competitively bind glucose transporters, limiting the uptake of glucose and other substrates such as anthocyanins, paving the way for a molecular rationalization of the observed effect.

## Conflicts of Interest

The authors declare no conflicts of interest.

## Peer Review

The peer review history for this article is available at https://www.webofscience.com/api/gateway/wos/peer‐review/10.1002/mnfr.70073.

## Supporting information



Supporting Information

Supporting Information

Supporting Information

## Data Availability

The data that support the findings of this study are available from the corresponding author upon reasonable request.
