# Supplementary material for: Preventive Effect of Upland Pigmented Potatoes Against LPS‐Induced Inflammation in THP‐1 Macrophages
Source: Mol Nutr Food Res. 2025 Apr 25;69(15):e70073. doi: 10.1002/mnfr.70073 (PMC12319466; doi:10.1002/mnfr.70073)
Supplement: Supplementary file 1 — Supporting Information [file MNFR-69-e70073-s001.pdf]

Supporting Information – Supplementary Methods

Extraction and fractionation

Briefly, 8 g of lyophilized potato powder were treated with 300 mL ethanol:acetone 2:1 *v/v* plus 1 mL of 25% NH_3 (pH 10.2) to precipitate glycoalkaloids and vigorously shaken for 3 h at room temperature. After a centrifugation at 4500 rpm at 4°C, the supernatants were acidified to pH 3.4 with 6N HCl, concentrated under vacuum and resuspended in 12 mL 100% ethanol. The obtained extracts were then cooled at 0°C for 16 h and centrifuged again at 4500 rpm at 4°C to separate the insoluble fraction. Extracts from Kennebec, Desirée and Bleuet were analyzed by spectrophotometric and HPLC-DAD analyses and then stored at -20°C until use.

The anthocyanin fraction (ACN fraction) was isolated from Bleuet extract via liquid-liquid extraction using a separatory funnel. Briefly, an aliquot of the Bleuet extract was diluted 3-fold in deionized H_2O and then sequentially treated with *n*-hexane (30 mL for 3 times) followed by ethyl acetate (30 mL for 4 times) to remove carotenoids and CGAs, respectively. The residual fraction was enriched in anthocyanins. Solvents were evaporated under vacuum and the isolated ACN fraction was resuspended in 100% ethanol and acidified at pH 3.4. The anthocyanin recovery with respect to the Bleuet extract was 89%. Hence, the concentrations used in cell experiments were determined based on the Pg3G equivalent content of the Bleuet extract or ACN fraction and the other extracts were diluted in culture medium using the same volumes.

Spectrophotometric and HPLC-DAD analyses of extracts and ACN fraction

The CGA content in Kennebec, Desirée and Bleuet extracts was analyzed by HPLC-DAD performed with a JASCO system equipped with a diode array detector (MD-2010 JASCO). The pump (PU-980 JASCO) was coupled with a quaternary gradient unit (LG-1580-02 JASCO). The analytical data were evaluated using a software-management system of chromatographic data (ChromNAV, Jasco, version 1.14.01). The separation was performed by a reversed phase, using a C18 Purospher Star 250 × 4 mm column. The flow rate was 0.6 mL/min, the injection volume 15 µL, and the oven temperature was 42°C. The mobile phase consisted of water with 0.5% of formic acid (solvent A) and acetonitrile acidified with 0.5% of formic acid (solvent B). The

gradient was as follows (A/B): 95/5 0-5 min, from 95/5 to 80/20 in 10 min, 80/20 for 5 min, from 80/20 to 55/45 in 10 min, 55/45 for 10 min, from 55/45 to 95/5 in 10 min, 95/5 for 9 min. Total analysis time was 59 min. Peak identification, with detection at 325 nm, was performed by the direct comparison with commercial standard CGA at known concentrations used to calibrate the system for the quantitative evaluation. Under these conditions, the retention time of CGA resulted 9.8 minutes (Fig. S1). As for the characterization of anthocyanins, all the extracts were subjected to the same chromatographic run as before, with the detection wavelength at 520 nm (Fig. S2).

The extract for the total carotenoid analysis was obtained by mixing 0.3 mL of the ethanol extract with the same volume of *n*-hexane/ethyl acetate 2:1 added of 0.1% BHT (butylated hydroxytoluene). To this solution, 0.5 mL of 20% NaCl was added and vortexed for 30 seconds. After a centrifugation of the mixture at 5000*g* at 4°C, the reading at the spectrophotometer was made on the clear upper phase, after three-fold dilutions with *n*-hexane. The total carotenoid content was measured spectrophotometrically at 441 nm, calculated using 2540 as molar absorbance of zeaxanthin and expressed as ppm (mg/L) of zeaxanthin equivalents.

The total anthocyanin content was measured with the pH-differential method, following the method by Giusti and Wrolstad (2001)[1]. Briefly, 1 mL of the extracts was treated separately with 4 mL of pH 1.0 buffer (25 mM KCl pH 1) or 4 mL of pH 4.5 buffer (400 mM sodium acetate pH 4.5) for 20 minutes at room temperature. The samples were thoroughly mixed and the absorbance was measured at 520 nm and 700 nm. The total anthocyanin content was calculated according to the equation:

Amount [mg g^{-1}] = ΔA × 468,84/15600 × dilution factor x final volume (mL)/sample weight (g) where ΔA: (A510-A700) pH1 – (A510-A700) pH4.5. The value 468,84 represents the molecular mass of Pg3G, whereas 15600 is its molar absorbance. Each extract was analyzed in triplicate and the anthocyanin content expressed as ppm (mg/L) of Pg3G equivalents.

RNA extraction and Real Time RT-PCR analysis

Total RNA was purified from cell lysate using Direct-Zol™ RNA MiniPrep Kit (Zymo Research, Irvine, CA, USA). 1 µg of RNA was reverse-transcribed with the iScript cDNA Synthesis Kit (Bio-Rad, Hercules, CA, USA) and Real-Time RT-PCR was performed with the QuantiNova SYBR Green Kit (Qiagen, Hilden, Germany) in a

CFX96™ Real-Time PCR detection system (Bio-Rad). Each transcript level was normalized against *GAPDH* and expressed as fold change over the unchallenged counterpart (- LPS). Primers are listed in Table S1. Data are presented as mean ± SEM of two biological replicates in triplicate.

Enzyme-linked immunosorbent assay (ELISA)

Cytokine concentrations in cell supernatants were detected using Human TNF-α, Human IL-1β and Human IL-6 DuoSet® ELISA Kits (R&D Systems, Minneapolis, MN, USA) following the supplier's instructions. The absorbance was measured at 450 nm using the EnSight® Multimode Plate Reader (PerkinElmer, Waltham, MA, USA). Results are expressed as mean ± SEM of concentration of the secreted cytokine (pg/mL) versus the intracellular total protein, extracted with a lysis buffer containing 20 mM Tris-HCl pH 6.5, 150 mM NaCl, 1mM EDTA, 0.5% Sodium Deoxycholate, 1% Triton X-100, 0.1% SDS and the Protease Inhibitor Cocktail (Sigma-Aldrich) and quantified with the Bio-Rad Protein Assay (Bio-Rad). Analysis was conducted in duplicate with four technical replicates.

Molecular Docking

The docking pipeline was performed using the Schrödinger Maestro release 2023-3 build 125 (Maestro, Schrödinger, LLC, New York, NY, 2023). Structural refinement was performed using the Protein Preparation Wizard tool[2]. Briefly, this process included adding missing atoms (including hydrogens), assigning appropriate protonation states to ionizable groups at a pH of 7.0 ± 1, and optimizing the orientations of hydroxyl and amide groups to enhance the hydrogen bond network. Additionally, energy minimization using the OPLS[3] force field was performed to resolve steric clashes and other unfavorable interactions.

Docking grids were designed for each transporter to include the entire substrate translocation channel. For GLUT-1, the grid dimensions were 26 Å × 37 Å × 24 Å, centered at coordinates (585.1031, -27.2976, 206.8556). For GLUT-3, the grid dimensions were 31 Å × 48 Å × 27 Å, centered at coordinates (-53.7029, 11.2736, 11.7294). For SGLT-1, the grid dimensions were 53 Å × 55 Å × 45 Å, centered at coordinates (134.5145, 130.0027, 131.8711).

The docking process was performed using GlideXP[4], enabling flexible ligand sampling.

Bidimensional protein-ligand interaction diagrams were generated using the Ligand Interaction tool from the Schrödinger Maestro suite, with a distance cutoff of 3 Å from the ligand atoms. Structural images were created using the open-source version of PyMOL (Schrödinger, LLC, The PyMOL Molecular Graphics System, Version 2.6).

References

[1] M. M. Giusti, R. E. Wrolstad, *Curr. Protoc. Food Anal. Chem.* **2001**, *00*, F1.2.1-F1.2.13.

[2] G. Madhavi Sastry, M. Adzhigirey, T. Day, R. Annabhimoju, W. Sherman, *J. Comput. Aided. Mol. Des.* **2013**, *27*, 221–234.

[3] K. Roos, C. Wu, W. Damm, M. Reboul, J. M. Stevenson, C. Lu, M. K. Dahlgren, S. Mondal, W. Chen, L. Wang, R. Abel, R. A. Friesner, E. D. Harder, *J. Chem. Theory Comput.* **2019**, *15*, 1863–1874.

[4] R. A. Friesner, R. B. Murphy, M. P. Repasky, L. L. Frye, J. R. Greenwood, T. A. Halgren, P. C. Sanschagrin, D. T. Mainz, *J. Med. Chem.* **2006**, *49*, 6177–6196.
