# Supplementary material for: Preventive Effect of Upland Pigmented Potatoes Against LPS‐Induced Inflammation in THP‐1 Macrophages
Source: Mol Nutr Food Res. 2025 Apr 25;69(15):e70073. doi: 10.1002/mnfr.70073 (PMC12319466; doi:10.1002/mnfr.70073)
Supplement: Supplementary file 2 — Supporting Information [file MNFR-69-e70073-s003.pdf]

Supporting Information – Supplementary Figures

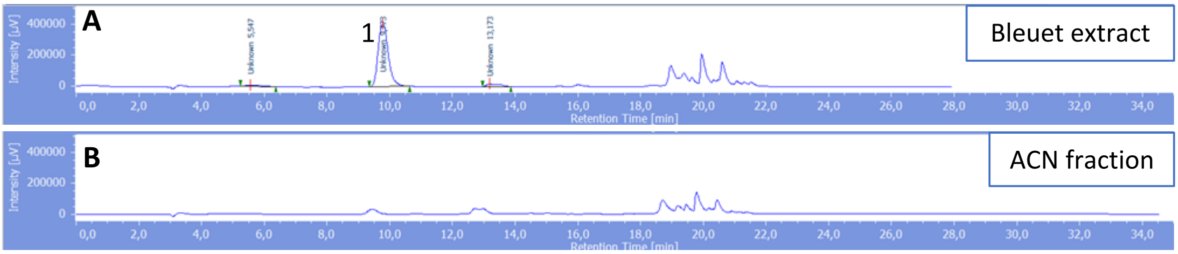

Peak number	Retention time (min)	Identity
1	9.8	Chlorogenic acid

Fig. S1 HPLC chromatogram with the identification of main phenolic acids present in Bleuet extract (**A**) and the isolated ACN fraction (**B**). Detection at 325 nm.

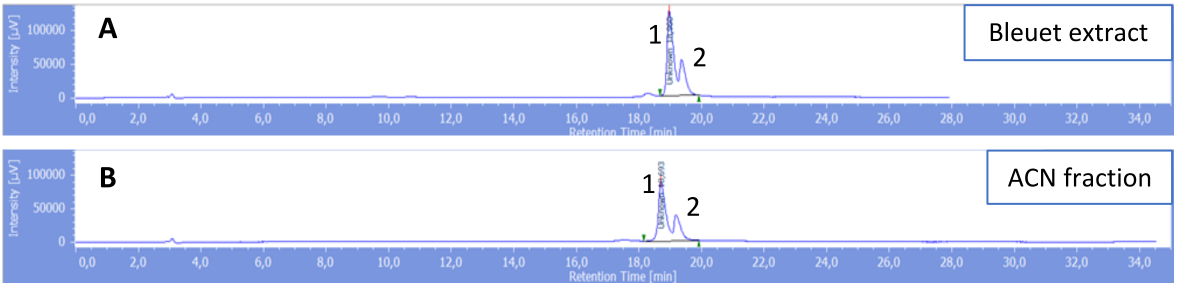

Peak number	Retention time (min)	Identity
1	18.7	Peonidin 3-rutinoside 5-(coumaroyl glucoside)
2	19.3	Pelargonidin 3-rutinoside 5-(coumaroyl glucoside)

Fig. S2 HPLC chromatograms with the characterization of main anthocyanins present in Bleuet extract (**A**) and its isolated ACN fraction (**B**). Detection at 520 nm.

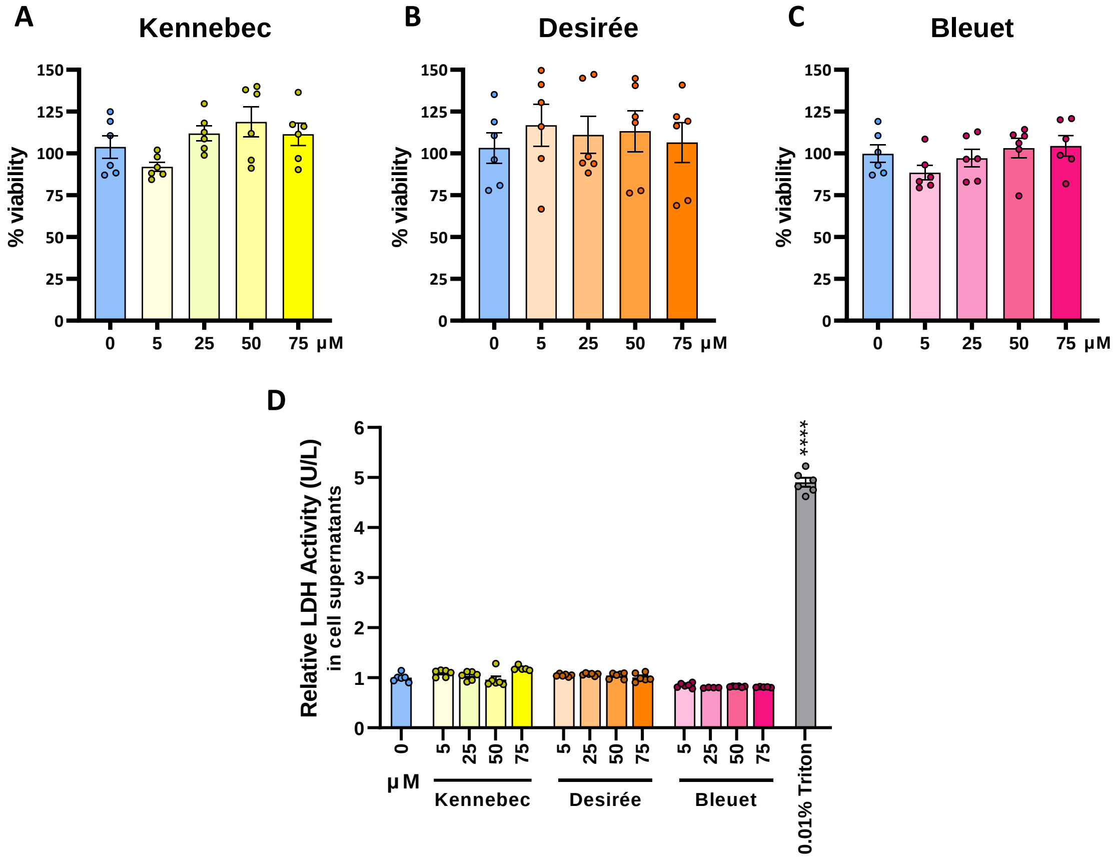

Fig. S3 Cell viability of THP-1 macrophages treated for 48h with increasing concentrations of Kennebec (**A**), Desiree (**B**) and Bleuet (**C**) extracts (0, 5, 25, 50 and 75 μM) was determined by MTT test and expressed as relative percentage of relative controls (0 μM) (**A-C**). Non-cytotoxicity of extracts was also verified measuring the LDH activity relative to the negative control (0 μM) in the cell medium. A positive control was used treating cells with 0.01% Triton X-100 (**D**). Results are the means ± SEM of three biological replicates in duplicate (n = 4-6). ****$p<0.0001$ indicate significant differences versus 0 μM.

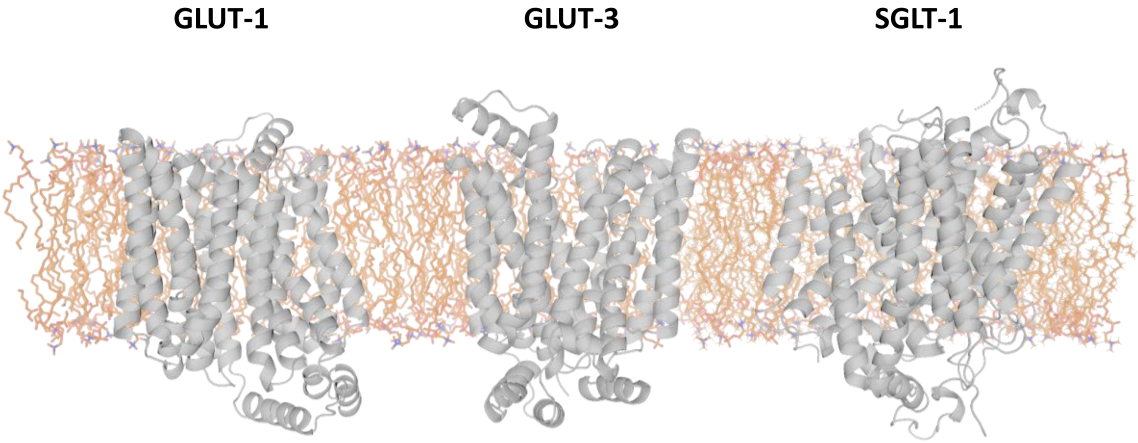

Fig. S4 GLUT-1, GLUT-3, and SGLT-1 structures. The three transporters are shown in gray cartoon and embedded in a POPC membrane generated with the CHARMM-GUI web server. The membrane is represented as sticks with carbon (C) atoms in light orange, phosphorus (P) atoms in orange, oxygen (O) atoms in red, and nitrogen (N) atoms in blue. The structures correspond to the PDB IDs 4PYP (GLUT-1), 4ZWC (GLUT-3), and 7SLA (SGLT-1).

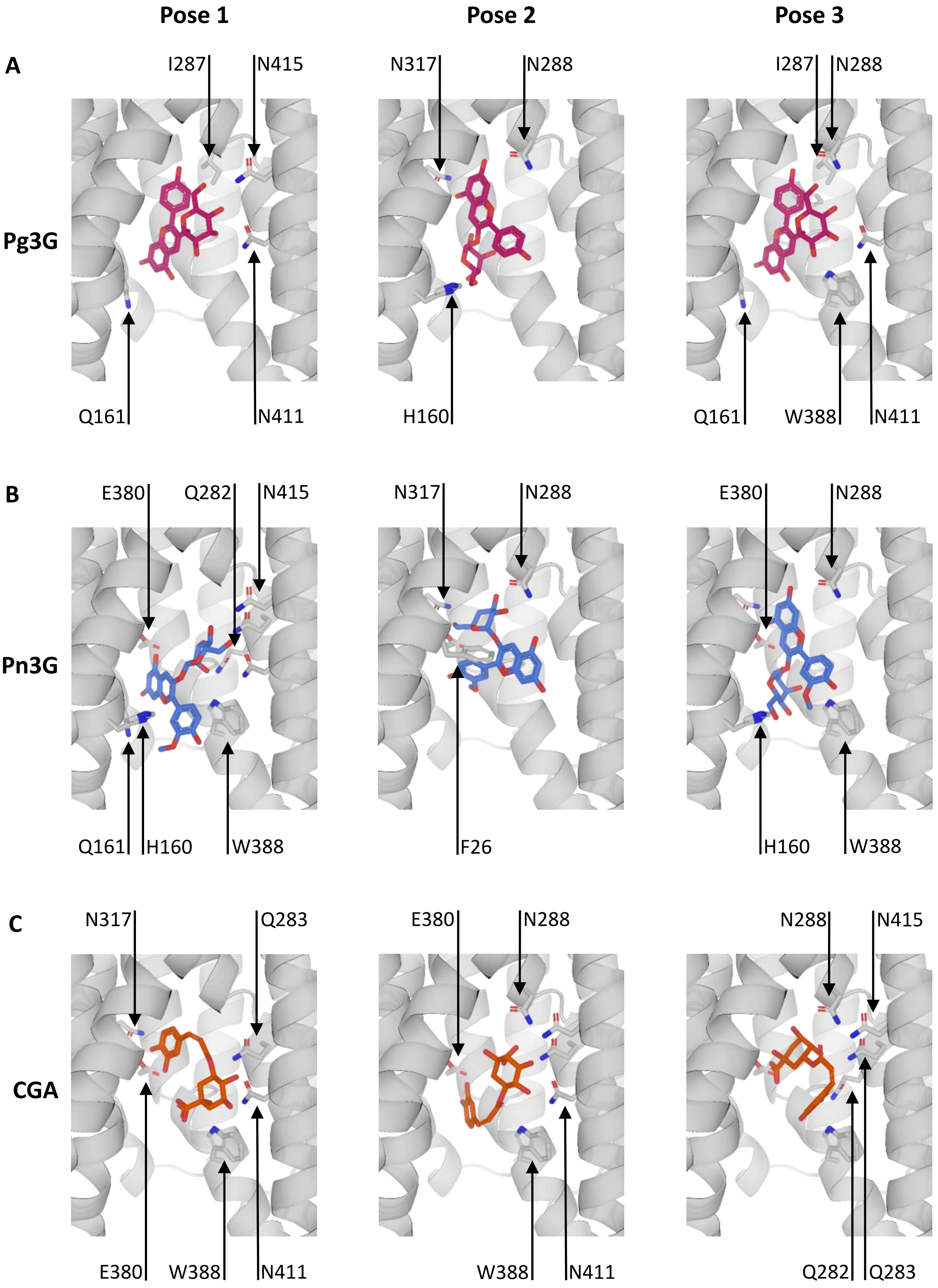

Fig. S5 Best docking poses targeting GLUT-1 protein, three-dimensional representation. Pg3G ligand is shown as stick with carbon (C) atoms in pink and nitrogen (N) atoms in red (**A**). Pn3G ligand is represented as stick with C atoms in blue and N atoms in red (**B**). CGA ligand is represented as stick with C atoms in orange and N atoms in red (**C**). Hydrogen atoms have been omitted for clarity. The transporter is represented in cartoon and colored in light gray. The relevant protein residues interacting with the ligand are represented in sticks with C atoms in light gray, oxygen (O) atoms in red, and N atoms in blue.

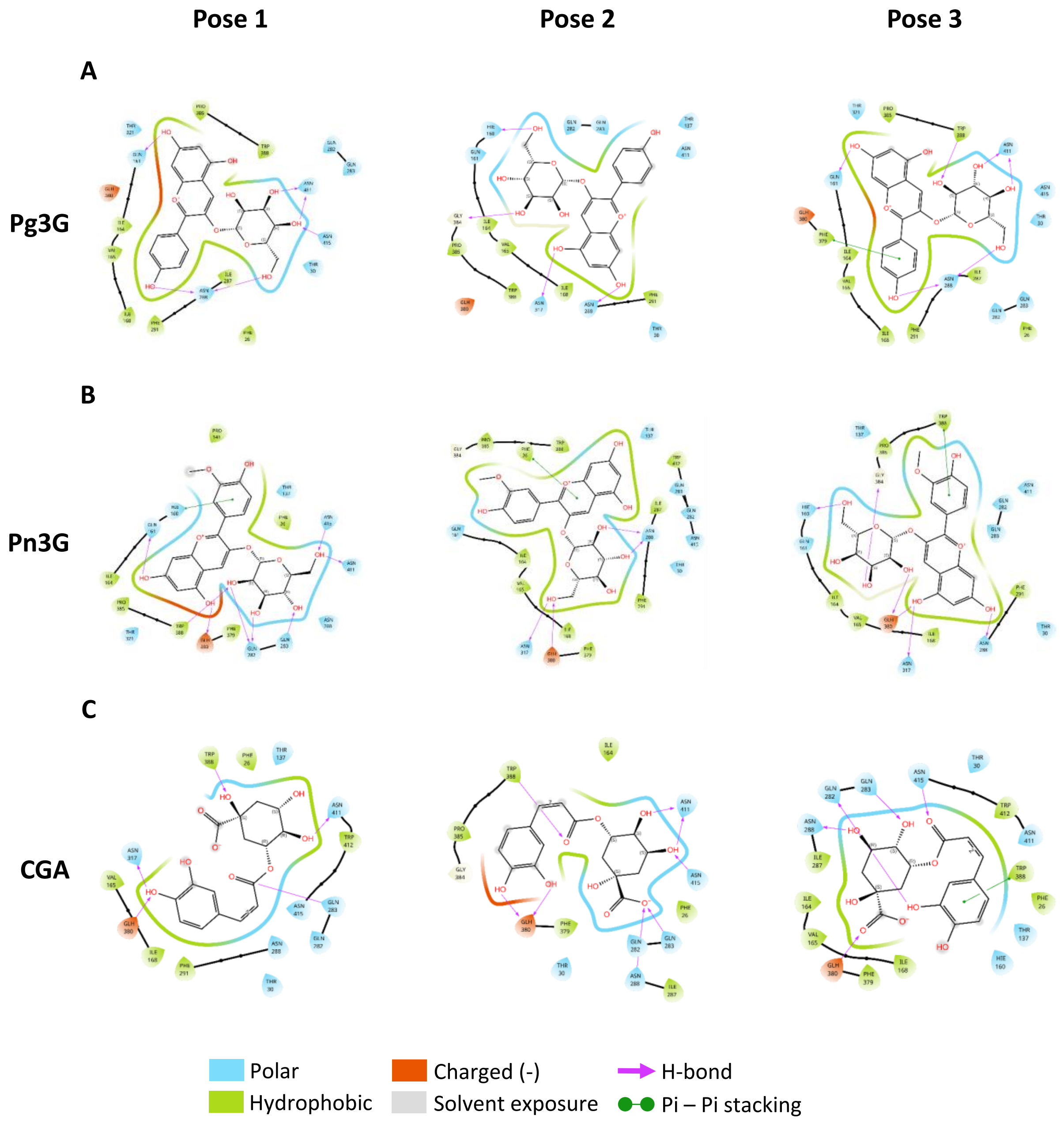

Fig. S6 Best docking poses targeting GLUT-1 protein, bidimensional protein-ligand interaction diagrams. Pg3G (**A**), Pn3G (**B**) and CGA (**C**) ligands.

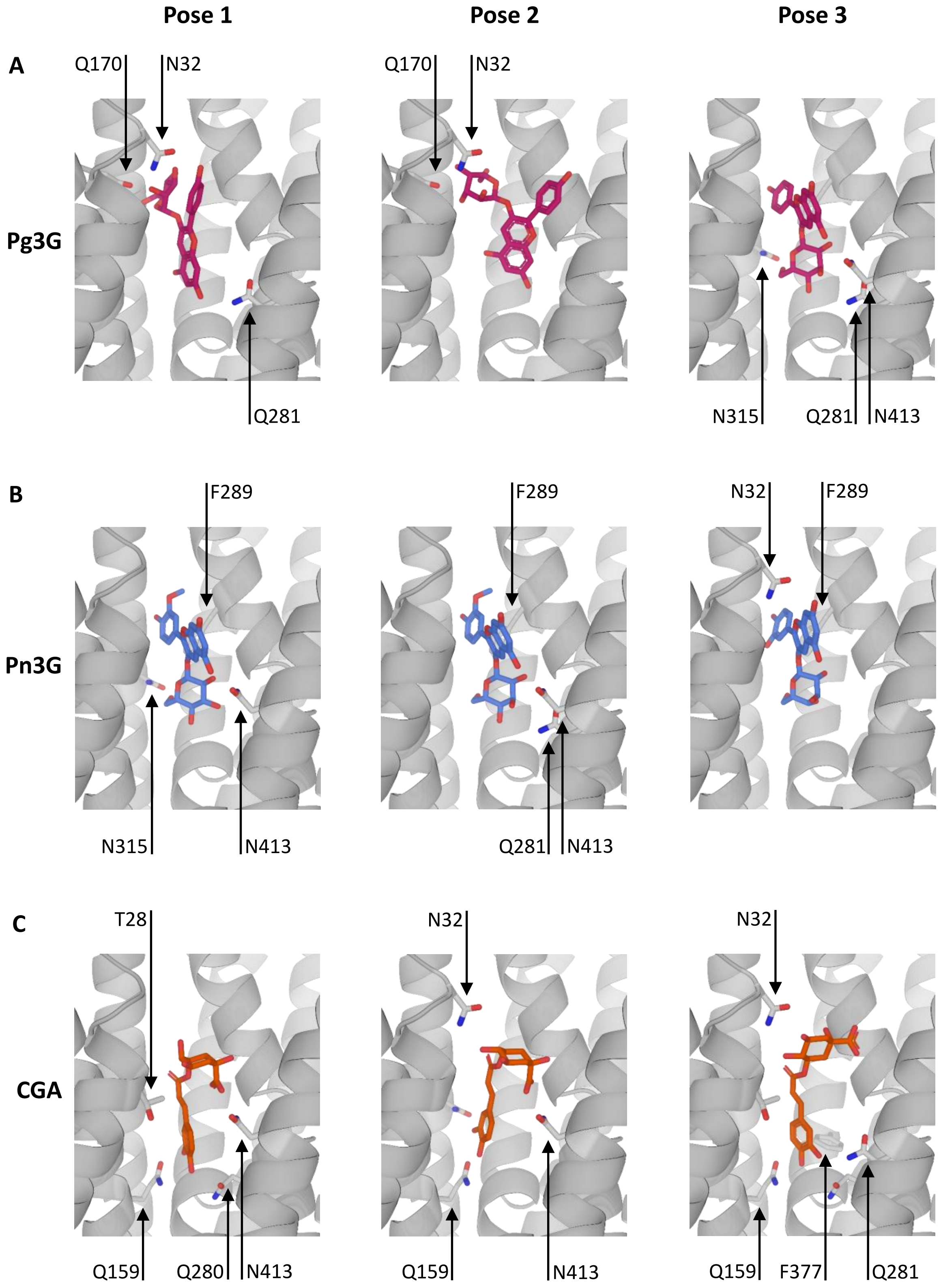

Fig. S7 Best docking poses targeting GLUT-3 protein, three-dimensional representation. Pg3G ligand is shown as stick with carbon (C) atoms in pink and nitrogen (N) atoms in red (**A**). Pn3G ligand is represented as stick with C atoms in blue and N atoms in red (**B**). CGA ligand is represented as stick with C atoms in orange and N atoms in red (**C**). Hydrogen atoms have been omitted for clarity. The transporter is represented in cartoon and colored in light gray. The relevant protein residues interacting with the ligand are represented in sticks with C atoms in light gray, oxygen (O) atoms in red, and N atoms in blue.

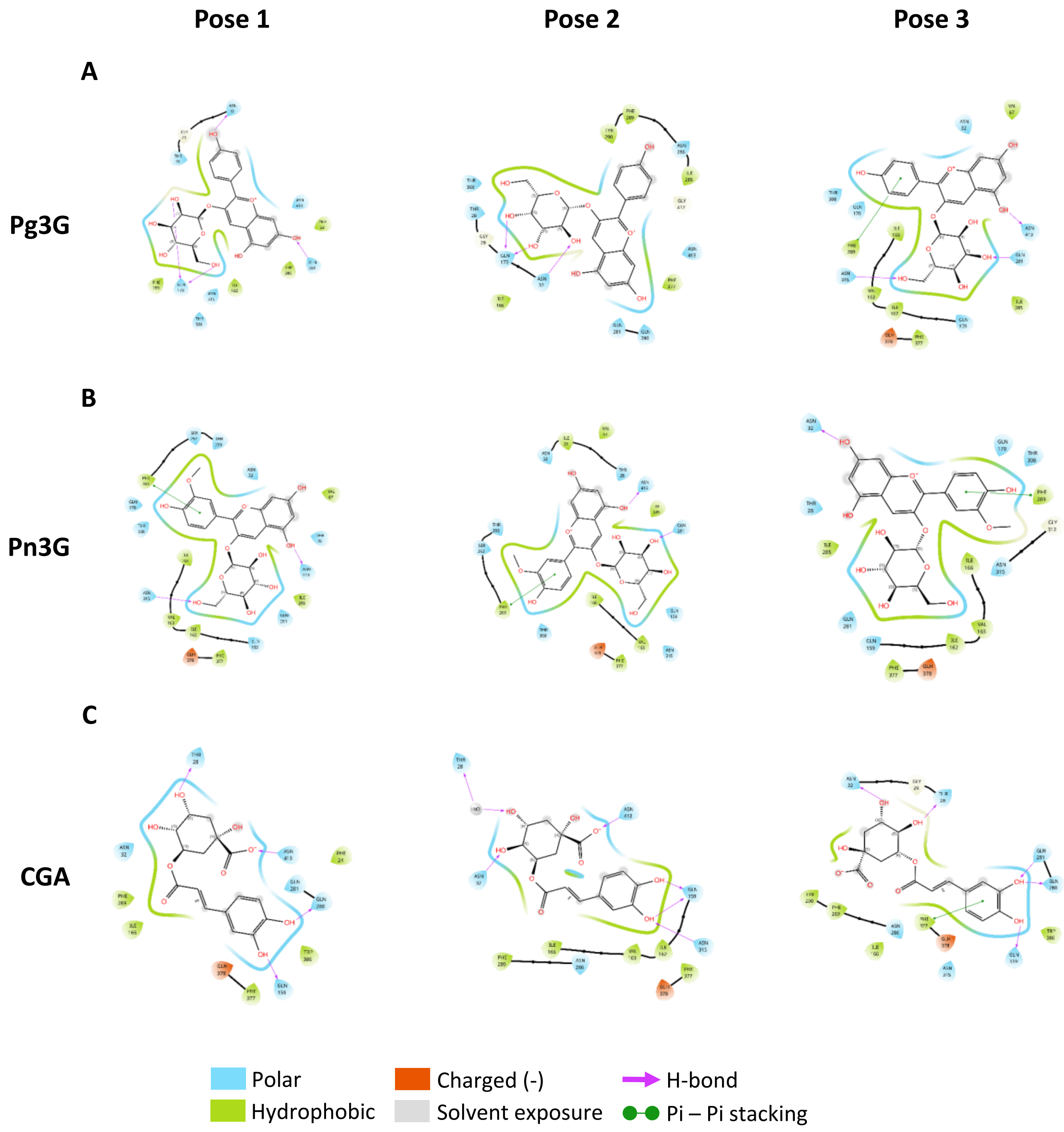

Fig. S8 Best docking poses targeting GLUT-3 protein, bidimensional protein-ligand interaction diagrams. Pg3G (**A**), Pn3G (**B**) and CGA (**C**) ligands.

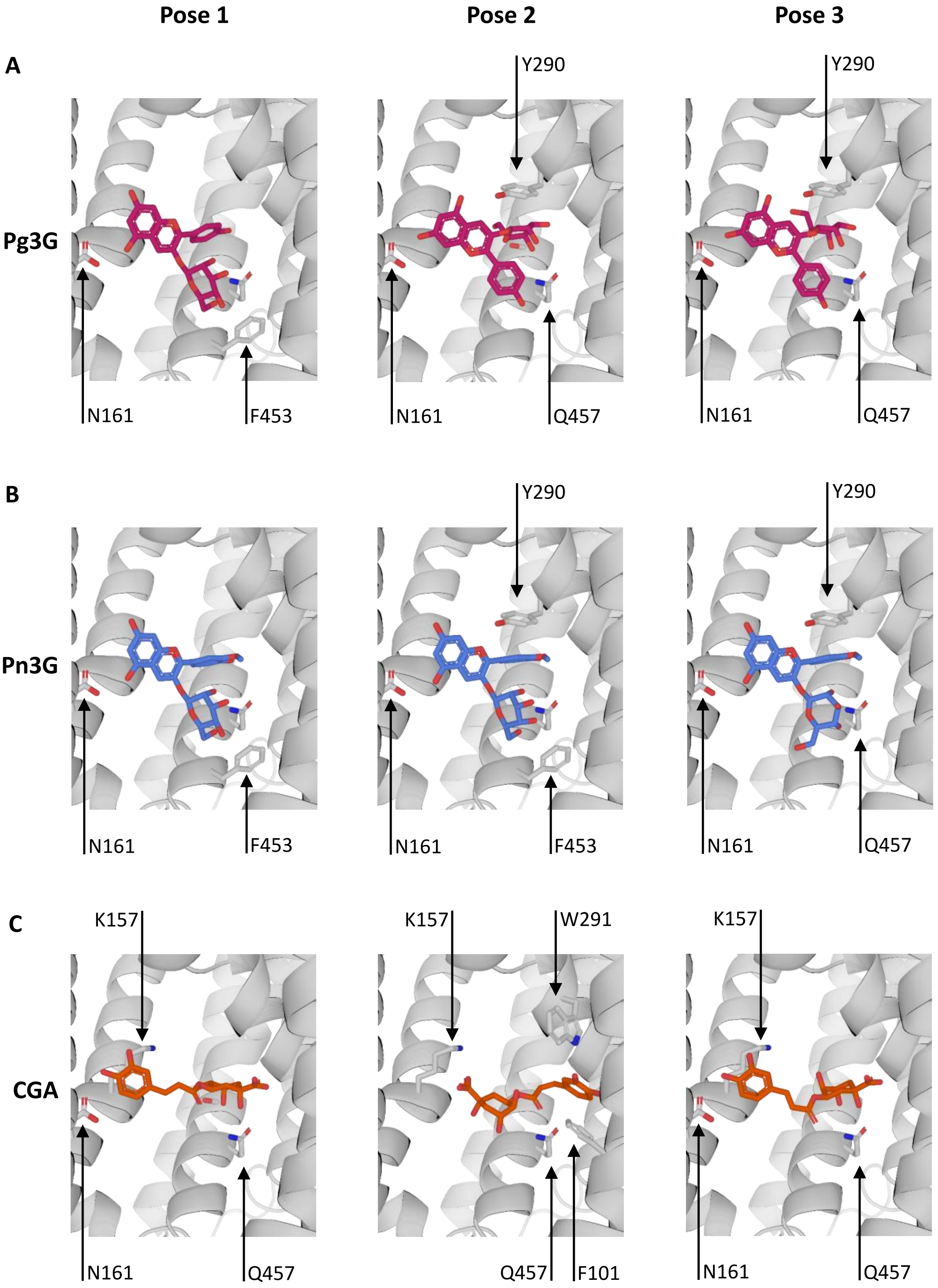

Fig. S9 Best docking poses targeting SGLT-1 protein, three-dimensional representation. Pg3G ligand is shown as stick with carbon (C) atoms in pink and nitrogen (N) atoms in red (**A**). Pn3G ligand is represented as stick with C atoms in blue and N atoms in red (**B**). CGA ligand is represented as stick with C atoms in orange and N atoms in red (**C**). Hydrogen atoms have been omitted for clarity. The transporter is represented in cartoon and colored in light gray. The relevant protein residues interacting with the ligand are represented in sticks with C atoms in light gray, oxygen (O) atoms in red, and N atoms in blue.

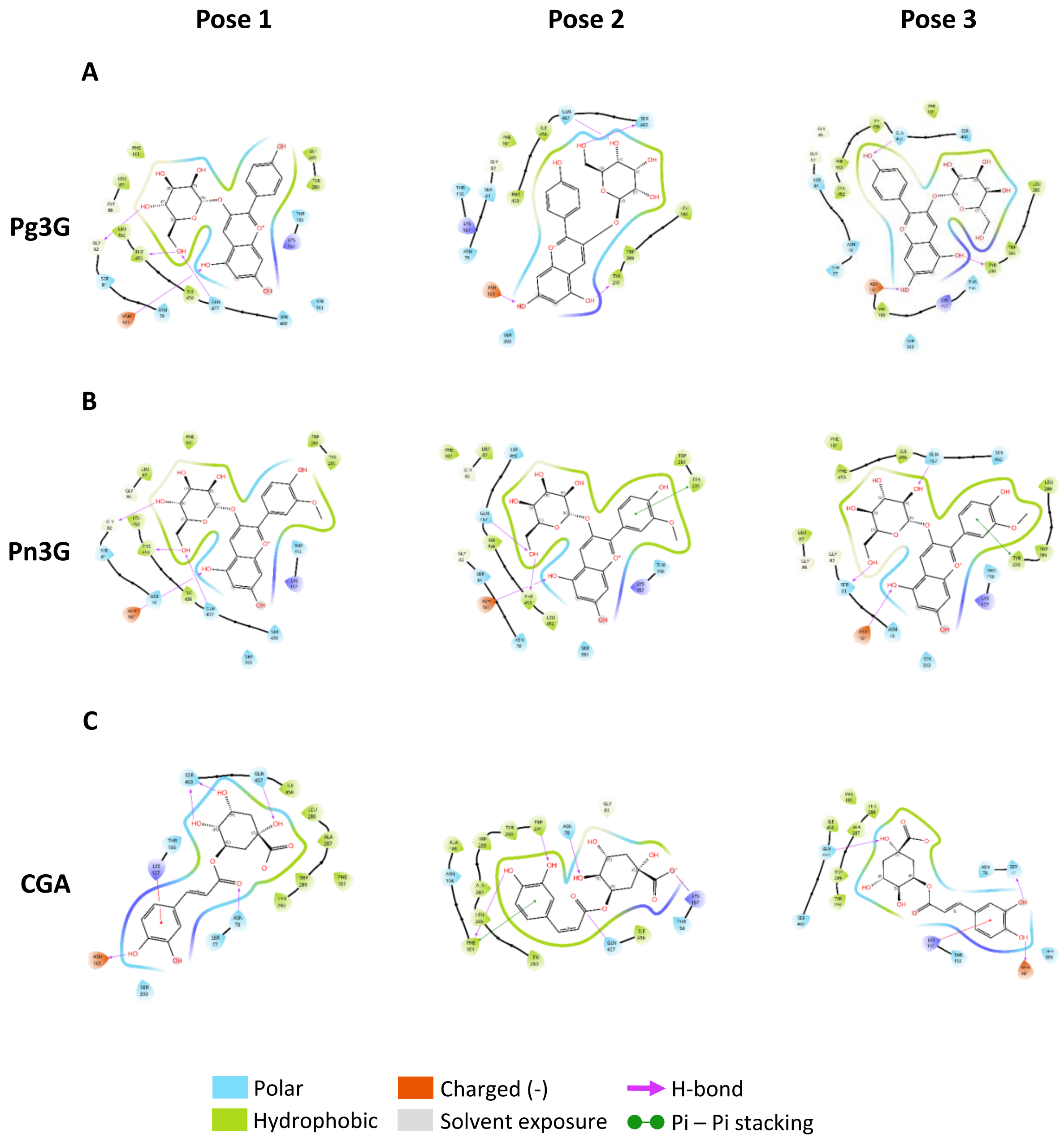

Fig. S10 Best docking poses targeting SGLT-1 protein, bidimensional protein-ligand interaction diagrams. Pg3G (**A**), Pn3G (**B**) and CGA (**C**) ligands.

A

Residues	β-NG	Pg3G			Pn3G			CGA		
	Control PDB ID 4PYP	Pose 1 -13.017	Pose 2 -12.810	Pose 3 -12.750	Pose 1 -14.210	Pose 2 -13.768	Pose 3 -13.391	Pose 1 -11.791	Pose 2 -11.726	Pose 3 -10.952
Phe 26						X				
His 160			X		X		X			
Gln 161		X		X	X					
Gln 282					X					X
Gln 283					X			X	X	X
Ile 287		X		X						
Asn 288	X	X	X	X		X	X		X	X
Asn 317	X		X			X	X	X		
Phe 379				X						
Glu 380					X	X	X	X	X	X
Gly 384			X				X			
Trp 388				X	X		X	X	X	X
Asn 411		X		X	X			X	X	
Asn 415		X			X				X	X

B

Residues	A-Mal	Pg3G			Pn3G			CGA		
	Control PDB ID 4ZWC	Pose 1 -9.738	Pose 2 -9.305	Pose 3 -9.089	Pose 1 -11.594	Pose 2 -11.082	Pose 3 -9.736	Pose 1 -10.371	Pose 2 -9.794	Pose 3 -9.476
Thr 28								X		X
Asn 32		X	X				X		X	X
Gln 159	X							X	X	X
Gln 170		X	X							
Gln 280	X							X		X
Gln 281	X	X		X		X				X
Phe 289				X	X	X	X			
Asn 315	X			X	X				X	
Phe 377										X
Trp 386	X									
Asn 413				X	X	X		X	X	

C

Residues	Pg3G			Pn3G			CGA		
	Pose 1 -8.979	Pose 2 -8.816	Pose 3 -8.571	Pose 1 -9.427	Pose 2 -9.240	Pose 3 -7.423	Pose 1 -12.719	Pose 2 -10.476	Pose 3 -10.329
Ser 77									X
Asn 78							X	X	
Ser 81						X			
Gly 82	X			X					
Phe 101								X	
Lys 157							X	X	X
Asp 161	X	X	X	X	X	X	X		X
Tyr 290		X	X		X	X			
Trp 291								X	
Phe 453	X			X	X				
Gln 457	X	X	X	X	X	X	X	X	X
Ser 460		X					X		

Fig. S11 Residue interactions observed in docking analysis. GLUT-1 (**A**), GLUT-3 (**B**) and SGLT-1 (**C**) residues involved in interactions with the ligands Pg3G, Pn3G, and CGA. An "X" indicates the presence of an interaction between the residue and the ligand in the corresponding pose. Residues in the "Control" column represent interactions observed with the ligand in the X-ray structure. Light yellow rows highlight amino acids shared among all the ligands in at least one pose; darker yellow rows shows residues in common between the ligands (at least one pose) and the control.
