# Supplementary material for: Preventive Effect of Upland Pigmented Potatoes Against LPS‐Induced Inflammation in THP‐1 Macrophages
Source: Mol Nutr Food Res. 2025 Apr 25;69(15):e70073. doi: 10.1002/mnfr.70073 (PMC12319466; doi:10.1002/mnfr.70073)
Supplement: Supplementary file 3 — Supporting Information [file MNFR-69-e70073-s002.pdf]

Supplementary Table 1. Primer sequences used for Real-Time RT-PCR.

**Primer**	**Sequence 5'-3'**
fw-TNF-α	CCTGGTATGAGCCCATCTATCT
rv-TNF-α	CAGGGCAATGATCCCAAAGT
fw-IL-1β	GGTGTTCTCCATGTCCTTTGTA
rv-IL-1β	GCTGTAGAGTGGGCTTATCATC
fw-IL-6	GGAGACTTGCCTGGTGAAA
rv-IL-6	CTGGCTTGTTCCTCACTACTC
fw-GAPDH	GCCTCAAGATCATCAGCAATGC
rv-GAPDH	CCACGATACCAAAGTTGTCATGG

fw: forward; rv: reverse
